# Lung and 'end organ' injury due to mechanical ventilation in animals: comparison between the prone and supine positions

**DOI:** 10.1186/cc4840

**Published:** 2006-02-28

**Authors:** George Nakos, Anna Batistatou, Eftychia Galiatsou, Eleonora Konstanti, Vassilios Koulouras, Panayotis Kanavaros, Apostolos Doulis, Athanassios Kitsakos, Angeliki Karachaliou, Marilena E Lekka, Maria Bai

**Affiliations:** 1 Department of Intensive Care Unit, University Hospital of Ioannina, Greece; 2 Department of Pathology, University of Ioannina, Greece; 3 Department of Anatomy-Histology-Embryology, University of Ioannina, Greece; 4 Department of Chemistry, University of Ioannina, Greece

## Abstract

**Introduction:**

Use of the prone position in patients with acute lung injury improves their oxygenation. Most of these patients die from multisystem organ failure and not from hypoxia, however. Moreover, there is some evidence that the organ failure is caused by increased cell apoptosis. In the present study we therefore examined whether the position of the patients affects histological changes and apoptosis in the lung and 'end organs', including the brain, heart, diaphragm, liver, kidneys and small intestine.

**Methods:**

Ten mechanically ventilated sheep with a tidal volume of 15 ml/kg body weight were studied for 90 minutes. Five sheep were placed in the supine position and five sheep were placed in the prone position during the experiment. Lung changes were analyzed histologically using a semiquantitative scoring system and the extent of apoptosis was investigated with the TUNEL method.

**Results:**

In the supine position intra-alaveolar hemorrhage appeared predominantly in the dorsal areas, while the other histopathologic lesions were homogeneously distributed throughout the lungs. In the prone position, all histological changes were homogeneously distributed. A significantly higher score of lung injury was found in the supine position than in the prone position (4.63 ± 0.58 and 2.17 ± 0.19, respectively) (*P *< 0.0001). The histopathologic changes were accompanied by increased apoptosis (TUNEL method). In the supine position, the apoptotic index in the lung and in most of the 'end organs' was significantly higher compared with the prone position (all *P *< 0.005). Interestingly, the apoptotic index was higher in dorsal areas compared with ventral areas in both the prone and supine positions (*P *< 0.003 and *P *< 0.02, respectively).

**Conclusion:**

Our results suggest that the prone position appears to reduce the severity and the extent of lung injury, and is associated with decreased apoptosis in the lung and 'end organs'.

## Introduction

Mechanical ventilation has constituted an indispensable part of basic life support in the intensive care unit for several decades and is undoubtedly essential for patients with acute lung injury/acute respiratory distress syndrome (ALI/ARDS). In recent years, however, it has become clear that mechanical ventilation can also be injurious. Repeated application of transalveolar pressures that exceed those corresponding to the inflation capacity causes tissue stresses and disrupts the lung. In animals, mechanical ventilation at high volumes and high pressures can cause ventilator-induced lung injury (VILI) with similar histological appearance to ALI/ARDS. These histological disorders are due to injury of the alveolar epithelium, basement membrane and microvascular endothelium and accompanied by high-permeability pulmonary edema. Injurious mechanical ventilation exacerbates the damage in previously injured lungs [1-3].

The damage to the lungs has been attributed to two overlapping mechanisms, namely mechanical damage of tissues and cells due to overdistention and shear stress (barotrauma or volutrauma) as well as mechanical damage due to the production, release and/or activation of cytotoxic and inflammatory cascades (biotrauma). In addition to inducing or worsening existing lung injury, the pulmonary production of inflammatory mediators is likely to spill over into the systemic circulation, also contributing to extrapulmonary end-organ failure [3,4].

Despite considerable progress, the death rate of patients with ALI/ARDS remains quite high [5]. In fact, most patients die from multisystem organ failure and not from hypoxia. However, pathogenesis of multiorgan failure in ARDS/ALI remains a dilemma. There is some evidence that multisystem organ failure is caused by increased apoptosis of the epithelial cells of 'end organs', such as the kidneys and small intestine [6,7]. Apoptosis is an active mechanism of cell death, which is important for the development and homeostasis of tissues. Environmental conditions or specific receptor/ligand interactions activate intracellular signaling pathways that lead to DNA cleavage and apoptotic cell death (for a review, see [8]).

As early as 1976 it was reported that placing patients with ALI/ARDS in the prone position improves their oxygenation [9-17]. Prone positioning improves secretion drainage from the airways, relieving lung compression by the heart and abdomen. The transalveolar forces are redistributed so as to allow expansion of the dorsal regions. All these events lead to an increase in end-respiratory lung volume, to better ventilation-perfusion matching and to alterations in chest-wall mechanics leading to regional changes in ventilation. The effects of prone ventilation on the cellular constituents of the lung alveoli have not so far been studied.

Our working hypothesis was that VILI can lead to distant organ damage through the increase in the circulation of mediators, including proapoptotic soluble factors, such as soluble Fas ligand [6]. In this respect, using injurious tidal-volume-induced lung damage, we studied the possible protective role of the prone position through the reduction of atelectasis and/or overdistention. In addition, we investigated whether cell apoptosis was related to the severity of tissue damage of the lung and other organs induced by mechanical ventilation.

## Materials and methods

### Animal preparation

Protocols were approved by the University of Ioannina animal research committee. We examined 10 sheep, each weighing 33 ± 5 kg. A peripheral vein was cannulated, and anesthesia was induced with katanine, maintained by continuous intravenous injection of midazolam and fentanyl citrate and paralyzed with pancuronium bromide. The animals were tracheotomized, and catheters were introduced into the carotid artery and the external jugular vein. Mechanical ventilation was provided with a Servo 900C ventilator (Siemens Elema, Solna, Sweden) in the volume control mode with a tidal volume of 15 ml/kg body weight for 90 minutes, with low positive end expiratory pressure (3 cmH_2_O) and with FiO_2 _of 0.5 in both groups. The respiratory rate was adjusted appropriately to maintain normocapnia at baseline measurements. Arterial pressure from the carotid artery and airway was recorded throughout the experiment. Blood gases, respiratory system compliance (calculated as the end-inspiratory airway pressure minus the end-expiratory pressure divided by the tidal volume) and biochemistry were measured before, during and at the end of the experiment. We continuously monitored the arterial blood pressure, the central venous pressure, the heart rate and the urine output. These parameters were kept stable by fluid infusion (normal saline). The animal temperature was also kept stable.

Five animals were placed in the supine position and five in the prone position during the whole experiment. The animals were exsanguinated at the end of the experiment, which lasted 90 minutes from the beginning of mechanical ventilation, while deeply anesthetized. The internal organs were removed and representative sections from the lungs, the brain, the heart, the diaphragm, the liver, the kidneys and the small intestine were taken and fixed in 10% buffered formalin.

### Histologic evaluation and TUNEL method

Paraffin sections, 5 μm thick, were stained with the standard H&E stain and examined using light microscopy. Lung changes were analyzed histologically using a semiquantitative scoring system, as previously described elsewhere [18]. Briefly, six slides – two from the upper lobe (one from the dorsal area and one from the ventral area), two from the lower lobe (one from the dorsal area and one from the ventral area) and two from the middle lobe in the right lung and the middle area in the left lung – were analyzed by two independent pathologists. The pathologists were blinded to the assignment of the animals. The slides were scanned in low power and the five fields with the most pronounced changes were chosen. The score given for each slide represented the mean score of these fields.

Four parameters were examined: alveolar fibrin edema, alveolar hemorrhage, septal thickening and intra-alveolar inflammatory cells. The changes were scored according to their extent (score 0, 1, 2 and 3 for an extent of 0%, <25%, 25–50% and >50%, respectively) and the severity of the injury (score 0 for no changes, score 1, 2 and 3 for more severe changes). The injury score represents the sum of the extent and the severity of injury.

Apoptosis was detected with the terminal deoxynucleotidyl-transferase-mediated dUTP nick end-labeling (TUNEL) method (Apo-tag kit; Oncor, Craithersburg, MD, USA) in 5 μm paraffin sections, as described in detail in previous studies [19,20]. Positive and negative controls were included in every staining. Positive staining in areas of lymphocytic infiltration served as the internal positive control. No staining was noted in negative controls.

Briefly, morphologically intact TUNEL-positive cells and apoptotic cells in H&E-stained studies were considered positive and are referred to as apoptotic cells. The number of apoptotic cells and apoptotic bodies was recorded by using the 40× objective lens, and at least 10 randomly selected fields were counted. The apoptotic index (AI) was expressed as the number of apoptotic cells/bodies per 10 high-power fields. Care was taken to avoid areas with extensive inflammation. The AI at the alveolar septum of the lungs, the neurons and glial cells, the muscle cells of the diaphragm, the hepatocytes, the glomerular and tubular renal cells, and the epithelial cells of the small intestinal epithelium were estimated.

### Statistical analysis

Statistical analysis was performed using the Statistical Package for Social Sciences (SPSS) version 12 for Windows (SPSS Inc., Chicago, Illinois, USA). Data were tested for normality with the Kolmogorov-Smirnov test and are presented as the mean ± SD. All variables were normally distributed. Comparisons between the prone and supine positions were made using a *t* test. Comparisons between the ventral and dorsal regions of the lungs in either the supine position or the prone position were made using a paired *t* test.

## Results

### Lung mechanics and blood gases

Lung mechanics and blood gas alterations and the biochemical data are presented in Tables 1 and 2, respectively. Blood gases and the compliance of the respiratory system deteriorated after 90 minutes of mechanical ventilation in both positions. The deterioration in blood gases as well as in the compliance due to VILI was significantly less prominent in the prone position. Transaminases (aspartate aminotransferase and alanine aminotransferase) increased during mechanical ventilation in the supine position, while they were both unchanged in the prone position. γ-Glutamyl transpeptidase, urea and creatinine were not altered during mechanical ventilation in both positions.

### ALI score in the prone and supine positions

**Figure 1:**
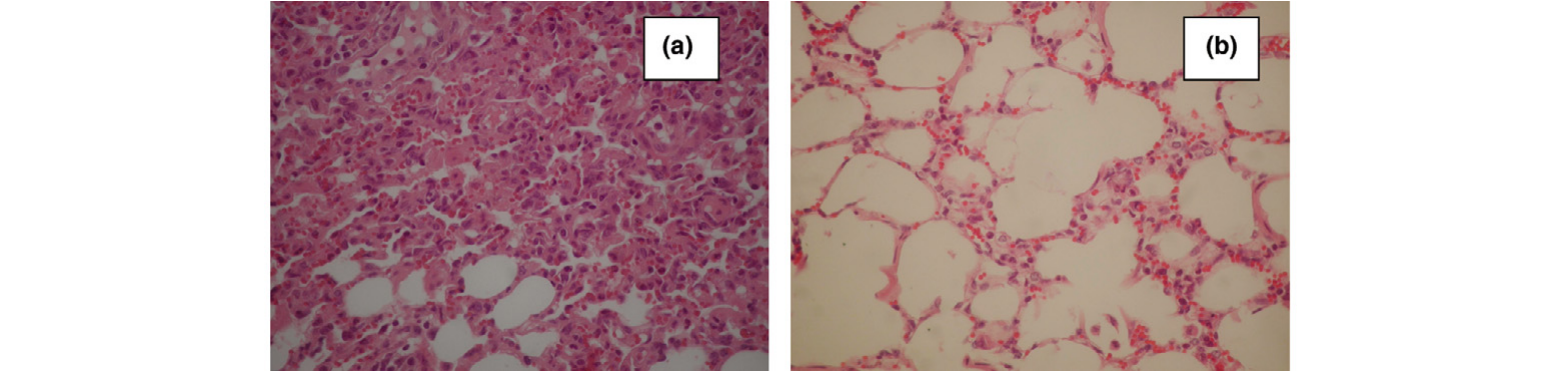
Histological changes of lungs (septal thickening, alveolar fibrin/edema, alveolar hemorrhage, intra-alveolar inflammatory cells) in animals placed in **(a)** the supine position and **(b)** the prone position (H&E, ×400).

In the lungs of the animals placed in the supine position the alveolar-septal membrane was thickened and there was considerable intra-alveolar edema and eosinophilic material. Furthermore, hemorrhage and increased numbers of inflammatory cells (lymphocytes, plasma cells, macrophages and polymorphonuclear neutrophil granulocytes) were observed (Table 3). Consolidated areas were frequently encountered (Figure 1a). In animals placed in the prone position the lung injury was milder (Table 3). There was considerably less inflammatory infiltration, alveolar edema, hemorrhage thickening of the alveolar-septal membrane and consolidation. In addition, many areas appeared uninjured or minimally affected (Figure 1b). The differences between the supine and prone positions were statistically significant (*P *< 0.0001). Interestingly, the overall histological findings for each animal were consistent in all lung areas – upper, middle and lower, ventral and dorsal (Table 3). When alveolar hemorrhage was considered alone, however, there was a significant difference between ventral and dorsal samples in animals placed in the supine position. In these animals the mean score for alveolar hemorrhage was 4.8 ± 0.84 in the ventral areas and was 2.6 ± 0.55 in the dorsal areas of both lungs (*P *< 0.01). This difference was not evident in animals placed in the prone position.

### Apoptotic index in the prone and supine positions

**Figure 2:**
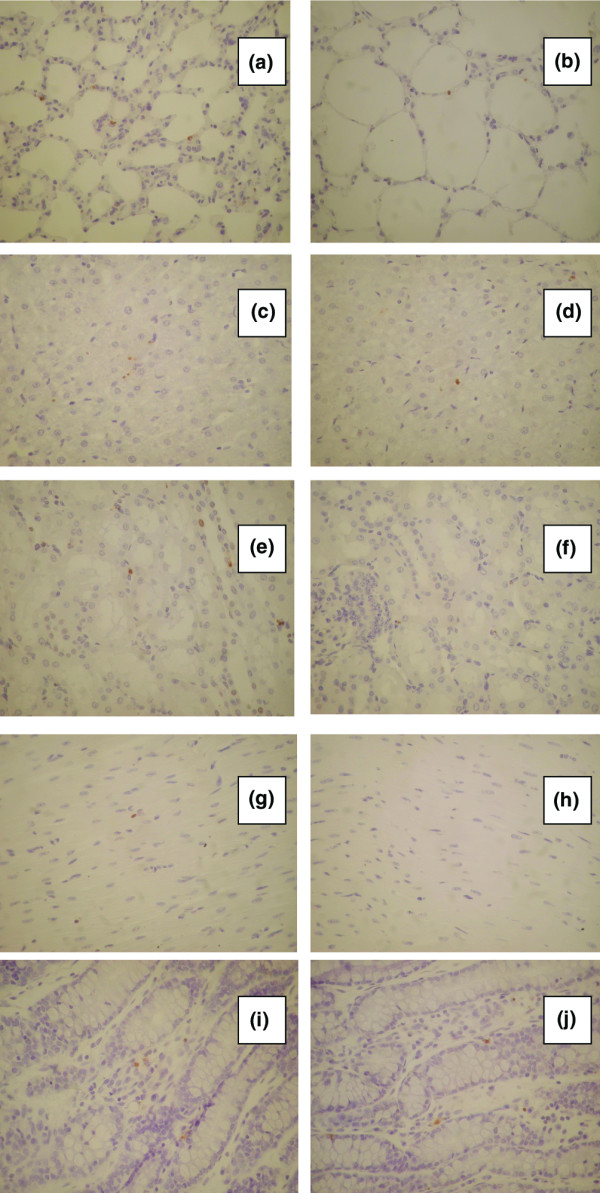
Apoptotic cells in the lungs [**(a)** supine position and **(b)** prone position], the liver [**(c)** supine position and **(d)** prone position], the kidneys [**(e)** supine position and **(f)** prone position], the diaphragm [**(g)** supine position and **(h)** prone position] and the small intestine [**(i)** supine position and **(j)** prone position] detected using the TUNEL method (×400).

TUNEL-positive nuclei/apoptotic bodies were observed in all animals in the lungs, and the AI was increased in the supine position group compared with the prone position group (Table 3 and Figure 2a,b). In both the supine position and the prone position, the mean value of the AI was higher in areas dorsal compared with ventral areas; the differences were statistically significant (*P *= 0.04 and *P *= 0.046, respectively). Moreover, the differences between the supine and prone positions were statistically significant in the dorsal lung areas as well in the ventral lung areas (*P *< 0.003 and *P *< 0.02, respectively) (Table 3).

The AI in the liver was far less than that in the lungs. The liver AI was increased in the supine position group (Figure 2c,d). The difference was statistically significant (*P *< 0.05) (Table 3).

In the kidneys, particularly at the medulla, the nuclei of tubular epithelial cells were TUNEL-positive without morphological characteristics of apoptosis and were not included in the estimation of the AI. Counts were performed at the cortex (Figure 2e,f). The mean values of the AI were higher in the supine position in comparison with the prone position, but the differences between the two groups did not reach statistical significance (Table 3).

An increased AI was also detected in the myocytes of the diaphragm (Figure 2g,h). The mean value of the AI was remarkably increased in the supine position compared with the prone position, and the difference was statistically significant (*P *< 0.001) (Table 3).

An increased AI was also detected in the epithelial lining of the small intestine villi and crypts in the supine position group compared with the prone position group. This difference was not statistically significant, however (Figure 2i,j and Table 3).

The AI in the brain was low in both the supine position and the prone position groups.

## Discussion

The main finding in this study was the reduction of the severity of and the extent of VILI in the prone position. This protective result of the prone position was associated with decreased cell apoptosis in the lung and other organs, including the liver and the diaphragm.

We have shown that mechanical ventilation in relatively high volumes causes injury to the lung parenchyma of animals, which can be detected and semiquantitated using light microscopy. These histologically defined changes were significantly more extensive in the supine position than in the prone position. Furthermore, intra-alveolar hemorrhage appeared predominantly in the dorsal areas in the supine position, while the other histologic changes (alveolar/fibrin edema, septal thickening, intra-alveolar inflammatory cells) were homogeneously distributed throughout the lungs. All the histological changes were homogenously distributed in the prone position.

The histologic changes in the lung were accompanied by an increased AI at the alveolar septum. It is interesting that the AI was significantly higher in dorsal areas compared with ventral areas in both the prone and supine positions. We also present evidence supporting the hypothesis that an injurious ventilatory strategy administered to the lungs can lead to damage of 'end organs', probably associated with apoptosis. Interestingly, the prone position appears to reduce the severity and the extent of the lung injury and is associated with a decreased AI in the lungs and 'end organs'. The deterioration in blood gases as well as in the respiratory system compliance was in accordance with the lung injury and was lower in the prone position. The increase of PCO_2 _in the supine position could be attributed to the increase of dead space due to lung injury and basal atelectasis. Hypercapnia has been considered as a protective factor rather than a harmful one in lung injury [21]. It was therefore not a factor favoring the deference observed between the supine and prone positions.

When first recognized, ALI/ARDS was considered a diffuse disease of the lungs and the injury was considered homogeneously distributed. Computed tomographic scanning has demonstrated that alveolar filling, consolidation and atelectasis occur predominantly in dependent lung zones, whereas other areas may be relatively spared [22-28]. Rouby and colleagues reported that the lung injury in ARDS is actually heterogeneous, with collapsed areas, areas of regional hyperinflation and normal areas [29]. Bronchoalveolar lavage studies indicate, however, that even radiographically spared, nondependent areas may have substantial inflammation [30]. Our histological findings indicate that the VILI in the supine position as well in the prone position affects the whole lung quite homogeneously, except for the hemorrhage in the supine position, which was higher in dependent areas of the lung. This phenomenon could be due to greater tissue stresses and shearing force induced by the inspiratory pressure in the dependent areas of the lung, which are most subject to closure. The hemorrhage was significantly less and was homogeneously distributed in the prone position. This fact is probably due to expansion of the dorsal regions resulting in a reduction of the shear stress [15,26,31,32].

Over the past decade VILI has emerged as a clinical issue [2,32,33]. The clinical importance of VILI has been documented in the ARDS Network study, where a reduction by 22% in the mortality of patients was noted when the mechanical load exerted on the lungs was reduced with lowering of the tidal volume [5]. Ventilation with high tidal volume results in the release of cytokines and other proinflammatory molecules [34]. In addition to inducing lung injury or worsening existing lung injury, this cascade of mediators may also contribute to extrapulmonary end-organ failure. Activation of the Fas/Fas ligand pathway in this process could be implicated as the apoptotic mechanism of the alveolar epithelium. Soluble Fas ligand, a main proapoptotic factor, is considered responsible for the increased apoptosis in 'end organs' [6,29,35,36].

Our results show that injurious mechanical ventilation increases the apoptosis in the lungs as well as in 'end organs'. These findings are consistent with those of Imai and colleagues [6], who demonstrated that the injurious mechanical ventilation can lead to epithelial cell apoptosis in organs distal to the lung, such as the kidneys. There is some evidence that increased apoptosis is accompanied by biochemical changes suggesting organ failure [6,7]. This could be an explanation for the high rates of multiple organ failure in patients with ARDS and the decrease in mortality when lung protective strategy is applied [5,6]. The role of apoptosis and necrosis in tissue injury and inflammation is not well understood, however. Serious lung injury could be accompanied by necrosis, while cell death in milder situations could be due to apoptosis [37].

The prone position, under the studied conditions, appears to decrease the severity and the extent of lung injury and is associated with a decrease in apoptosis of lung and 'end organ' tissues. Broccard and colleagues have also shown in animal models that, for the same pattern of ventilatory pressures, the prone position protects better against VILI [15]. It is known that the prone position improves oxygenation by quite complex mechanisms: Changes in lung recruitment are definitely one parameter contributing to improved lung oxygenation. Lung perfusion and alveolar ventilation are more uniformly distributed in the prone position compared with the supine position [15,22,38,39]. Our data provide another piece in the puzzle of ventilation-induced injury of lung and 'end organs'. We propose that although there might be no regional distribution in lung perfusion, there are definitely differences in vascular damage, leading to preferential intra-alveolar hemorrhage in the dorsal lung areas, particularly in animals in the supine position. Pronation ameliorates these differences. From a theoretical standpoint, shear stresses at the junction of open tissue and closed tissue will rise to high levels that may mechanically disrupt epithelial as well as endothelial membranes [30].

## Conclusion

Further studies should be conducted to clarify the role of prone ventilation on reducing oxygen toxicity, limiting VILI and possibly leading to increased overall survival. In our study the prone position appears to decrease the severity and the extent of the lung injury and is associated with decreased apoptosis in the lung and 'end organs'.

## Limitations and clinical applications

The main limitations of this study are that the measure of soluble pre-apoptotic and apoptosis-inducing factors was not possible and that only a single method (TUNEL) was used to confirm apoptosis. TUNEL is a widely used method to identify apoptotic cells *in vivo*. It is true that it has disadvantages, but when supported by the light microscopic analysis of cell morphology (as in this study) TUNEL is accepted in the literature for the detection of apoptotic cell death. The number of animals was quite small, but the variability (standard deviation) in each group of data was low enough to detect significant differences. Furthermore, the conclusions of this study are limited to the use of a high tidal volume in noninjured lungs for a short period of time.

The way we ventilate patients is critical to their outcomes, and it is of high importance to focus on using gentle ventilatory strategies in order to minimize VILI. A low tidal volume aids in reducing the ventilator lung injury but it can also result in dependent atelectasis. A positive end expiratory pressure above the inflection point might attenuate this problem, and lead to overdistention of the nondependent region [40]. A combination of the prone position with a low tidal volume and an optimal positive end expiratory pressure could be a meaningful strategy to minimize VILI. Furthermore, it is conceivable that at some point in the future we will be focusing on inhibition of apoptosis with antimediator therapy.

The apparent 'clinical implication' of this study is that using an excessively high tidal volume for even a short period of time can have dramatic consequences on lung morphology and function, and might be sufficient to induce cascades finally leading to nonpulmonary organ damage. Beside that, even the application of a modest tidal volume in injured lung with an inhomogeneous distribution could result in local damage.

## Key messages

• 	The utilization of an excessively high tidal volume for even a short period of time can have dramatic consequences on lung morphology and function, and might be sufficient to induce nonpulmonary organ damage.

• 	The prone position appears to decrease the severity and the extent of the lung injury.

• 	The prone position is associated with decreased apoptosis in the lung and 'end organs'.

## Abbreviations

AI = apoptotic index; ALI = acute lung injury; ARDS = acute respiratory distress syndrome; FiO_2 _= fraction inspired oxygen; H&E = haematoxylin–eosin; PCO_2 _= partial pressure of CO_2_; TUNEL= terminal deoxynucleotidyl-transferase-mediated dUTP nick end-labeling; VILI = ventilator-induced lung injury.

## Competing interests

The authors declare that they have no competing interests.

## Authors' contributions

GN, AB, PK, MEL and MB were involved in the design of the study. GN and AB wrote the final manuscript. GN performed the statistical analysis. AB, PK and MB participated in the histological studies and measurement of the AI. EG, NK, BK, AD, AKi, AKa and MEL participated in the animal preparation. All authors read and approved the final manuscript.

## Figures and Tables

**Table 1:** Gas exchange, respiratory system compliance and hemodynamics

	Supine position	Prone position	*P *value	95% confidence interval of the difference
PO_2_/FIO_2 _(mmHg)				
Baseline	416 ± 23.6	412.4 ± 25.5	NS	
90 minutes	105.6 ± 24.1	251.6 ± 56.1	<0.001	-208.9 to -83.0
*P *value	<0.0001	<0.004		
95% confidence interval of the difference	272.8–247.9	84.8–236.7		
PCO_2 _(mmHg)				
Baseline	38.8 ± 1.8	40.8 ± 1.3	NS	
90 minutes	57.2 ± 1.5	43.0 ± 1.2	<0.001	2.2 to 6.1
*P *value	<0.002	<0.04		
95% confidence interval of the difference	-5.0 to -6.9	-1.1 to -5.8		
pH				
Baseline	7.408 ± 0.013	7.398 ± 0.008	NS	
90 minutes	7.322 ± 0.019	7.382 ± 0.018	0.0009	-0.08 to -0.03
*P *value	0.0005	NS		
95% confidence interval of the difference	0.063–0.108			
Static compliance of respiratory system (ml/cmH_2_O)				
Baseline	30.4 ± 3.8	25.9 ± 2.1	NS	
90 minutes	18.2 ± 2.8	22.8 ± 2.3	<0.02	-8.3 to -0.86
*P *value	<0.001	<0.003		
95% confidence interval of the difference	-10.1 to -14.3	-1.7 to -4.5		
Blood pressure (mmHg)				
Baseline	81.80 ± 7.294	85.60 ± 9.476	NS	
90 minutes	84.20 ± 5.167	86.00 ± 9.670	NS	
*P *value	NS	NS		
95% confidence interval of the difference				
Heart rate (beats/minutes)				
Baseline	117.2 ± 9.365	122.2 ± 6.140	NS	
90 minutes	130.4 ± 4.722	132.8 ± 5.891	NS	
*P *value	0.0074	0.0007		
95% confidence interval of the difference	-20.51 to -5.887	-13.72 to -7.484		

Static compliance of respiratory system = (end inspiratory airway pressure – end-expiratory pressure)/tidal volume.

**Table 2:** Biochemistry at the beginning and the end of experiment

	Supine position	Prone position	*P *value
Urea (mg/dl)			
Baseline	34.9 ± 11.5	43.4 ± 6.5	NS
90 minutes	41.1 ± 7.3	37.1 ± 8.4	NS
*P *value	NS	NS	
Creatinine (mg/dl)			
Baseline	0.62 ± 0.1	0.48 ± 0.11	NS
90 minutes	0.55 ± 0.08	0.53 ± 0.1	NS
*P *value	NS	NS	
aspartate aminotransferase (IU/l)			
Baseline	94 ± 21	98 ± 25	NS
90 minutes	147 ± 19	84 ± 27	<0.05
*P *value	<0.05	NS	
alanine aminotransferase (IU/l)			
Baseline	14 ± 6	16 ± 7	NS
90 minutes	27 ± 8	15 ± 9	<0.05
*P *value	<0.05	NS	
γ-Glutamyl transpeptidase (IU/l)			
Baseline	26 ± 18	29 ± 24	NS
90 minutes	33 ± 22	25 ± 23	NS
*P *value	NS	NS	

**Table 3:** Acute lung injury score and apoptotic index in the supine and prone position

	Supine position	Prone position	*P *value	95% confidence interval
Acute lung injury score	4.63 ± 0.58	2.17 ± 0.19	<0.0001	-3.9 to -1.82
Apoptotic index				
Lung dorsal	112 ± 22	45.6 ± 28	0.003	-103.6 to -29.78
Lung ventral	80 ± 28	35 ± 22	0.02	-82.6 to -8.1
*P *value	0.04	0.046		
95% confidence interval	2.37 to 61.09	0.29 to 20.5		
Liver	56 ± 21	23 ± 10	0.05	-66.78 to -7.8.1
Kidney	31 ± 14	17 ± 10	NS	
Small intestine	22 ± 11	16 ± 11	NS	
Diaphragm	10 ± 0.5	0.5 ± 0.4	0.001	-10.6 to -9.01

Acute lung injury score corresponds to the sum of the extent (score 0, 1, 2 and 3 for an extent of 0%, <25%, 25–50% and >50%) and the severity of lung injury (score 0 for no changes, score 1, 2 and 3 more severe changes). The apoptotic index was expressed as the number of apoptotic cells/bodies per 10 high-power fields.
